# Eccentric Viewing Training for Age-Related Macular Disease: Results of a Randomized Controlled Trial (the EFFECT Study)

**DOI:** 10.1016/j.xops.2023.100422

**Published:** 2023-10-31

**Authors:** Gary S. Rubin, Michael D. Crossland, Hannah M.P. Dunbar, Graham M. Brown, Bledi Petriti, Hannah Roche, Sarah V. Sirrell, Kavitha Thayaparan Broom, Robin D. Hamilton

**Affiliations:** NIHR Biomedical Research Centre for Ophthalmology at Moorfields Eye Hospital, London, UK

**Keywords:** Low vision, Rehabilitation, Eccentric viewing, Reading, Age-related macular disease

## Abstract

**Purpose:**

Eccentric viewing training for macular disease has been performed for > 40 years, but no large studies including control groups have assessed the benefits of this training. The EFFECT (Eccentric Fixation From Enhanced Clinical Training) study is a large randomized controlled trial of 2 types of eccentric viewing training.

**Design:**

Randomized controlled trial.

**Participants:**

Two hundred adults with age-related macular disease.

**Methods:**

Participants were randomized to either of the following: (1) a control group; (2) a group receiving supervised reading support; (3) a group receiving 3 sessions of training to optimize the use of their own preferred retinal locus; or (4) a group receiving 3 sessions of biofeedback training of a theoretically optimal trained retinal locus. All participants received standard low-vision rehabilitation.

**Main Outcome Measures:**

The primary outcome was patient-reported visual task ability measured on the Activity Inventory instrument at goal level. Secondary outcomes included reading performance and fixation stability.

**Results:**

There was no difference between groups on change in task ability (*F*_(3,174)_ = 1.48, *P* = 0.22) or on any of the secondary outcome measures. Visual acuity and contrast sensitivity fell in all groups, suggesting that disease progression outweighed any benefit of training.

**Conclusions:**

Eccentric viewing training did not systematically improve task ability, reading performance, or fixation stability in this study. Our results do not support the routine use of eccentric viewing training for people with progressing age-related macular disease, although this training may help people with end-stage disease. Rehabilitation of an inherently progressive condition is challenging.

**Financial Disclosure(s):**

Proprietary or commercial disclosure may be found in the Footnotes and Disclosures at the end of this article.

For people with macular disease and central vision loss, noncentral retina must be used to see. If one retinal area is used in place of the damaged fovea, it is known as the preferred retinal locus (PRL). The PRL usually develops within 6 months of vision loss in the second affected eye^1^ and can be in any quadrant of retina.^2^, ^3^, ^4^ Visual acuity^5^ and reading ability^6^ are not necessarily better at the PRL than other retinal areas, leading some to suggest that the PRL’s major purpose is a reference for the oculomotor system.^7^^,^^8^

It is theoretically better to use a PRL in superior or inferior retina (rather than nasal or temporal retina), to avoid vision being constrained horizontally by the physiological blind spot at the optic disc. In control subjects with simulated scotomas, horizontally placed PRLs adversely affect reading,^9^ and reading is fastest in the inferior retina,^10^ a region which also has the highest photoreceptor density^11^ and best attentional deployment.^12^

Fixation is less stable when using noncentral retina.^13^ Reduced fixation stability is associated with poorer visual acuity^14^, ^15^, ^16^, ^17^and worse reading ability.^18^, ^19^, ^20^ Some clinicians recommend eccentric viewing training for people with macular disease to reinforce the use of the PRL and to improve fixation stability.^21^, ^22^, ^23^ This sometimes involves developing a new, trained retinal locus (TRL), usually in superior retina, above the scotoma,^24^^,^^25^ or the part of the retina with best visual acuity.^26^ Training can change the PRL location,^26^ although this may have a negative effect on reading performance.^25^

There is not a strong evidence base for eccentric viewing training. Some studies have shown training can improve reading speed, visual acuity and fixation stability,^23^^,^^27^, ^28^, ^29^, ^30^, ^31^ but these studies are confounded by the lack of a control group, data being collected by the trainers themselves, poor randomization or simultaneous prescription of low vision aids, spectacles, and other rehabilitative strategies.^32^ A 2014 systematic review of eccentric viewing training found only 2 of 34 trials reached the Grading of Recommendations, Assessment, Development and Evaluation criterion^33^ of “moderate” quality, with 6 being of “low” quality and the remaining 26 being “very low” quality.^32^ One "moderate quality" paper showed a small improvement in reading speed of 27 words per minute after six 2-hour training sessions (within the test-retest variability of many reading speed tests).^34^ The other moderate quality trial showed an improvement in near visual acuity and activities of daily living in participants who received 8 sessions of computer-based eccentric viewing training. Improvements were also seen in those receiving magnification only, although PRL training was superior for “active” activities of daily living such as cooking, grooming, bathing, and housework.^35^

Here, we present the results of the EFFECT trial (Eccentric Fixation From Enhanced Clinical Training), a large, randomized controlled trial comparing 2 models of eccentric viewing training: training at the PRL and biofeedback training at a TRL. We included 2 control groups: 1 group who received supervised reading practice (as a criticism of previous research is unequal contact time between the control and intervention arms), and 1 without any intervention.

Our primary outcome was task ability measured using the Activity Inventory (AI), an adaptive visual function questionnaire widely used in low vision research.^36^, ^37^, ^38^, ^39^, ^40^, ^41^, ^42^, ^43^, ^44^ Secondary outcomes were reading performance and fixation stability.

## Methods

Participants were recruited from medical retina and low vision clinics at Moorfields Eye Hospital, London. All had a primary diagnosis of age-related macular degeneration (AMD), visual acuity of 6/12 to 3/60 (20/40 to 20/400, 0.3 logarithm of the minimum angle of resolution [logMAR] to 1.30 logMAR), and ≥ 1 point of absolute scotoma (< 0 decibels [dB]) identified on microperimetry. People who had previously received eccentric viewing training, who had serious hearing loss, or significant cognitive impairment (determined by the recruiting researcher), were excluded.

People with ocular comorbidity which may affect visual function were excluded. This was determined by the lead ophthalmologist (R.D.H.) on a case-by-case basis. People who had visually insignificant cataract, who had had simple cataract surgery, or who had diabetes with minimal retinopathy were included. Those who had received retinal surgery or with glaucoma were excluded.

**Figure 1:**
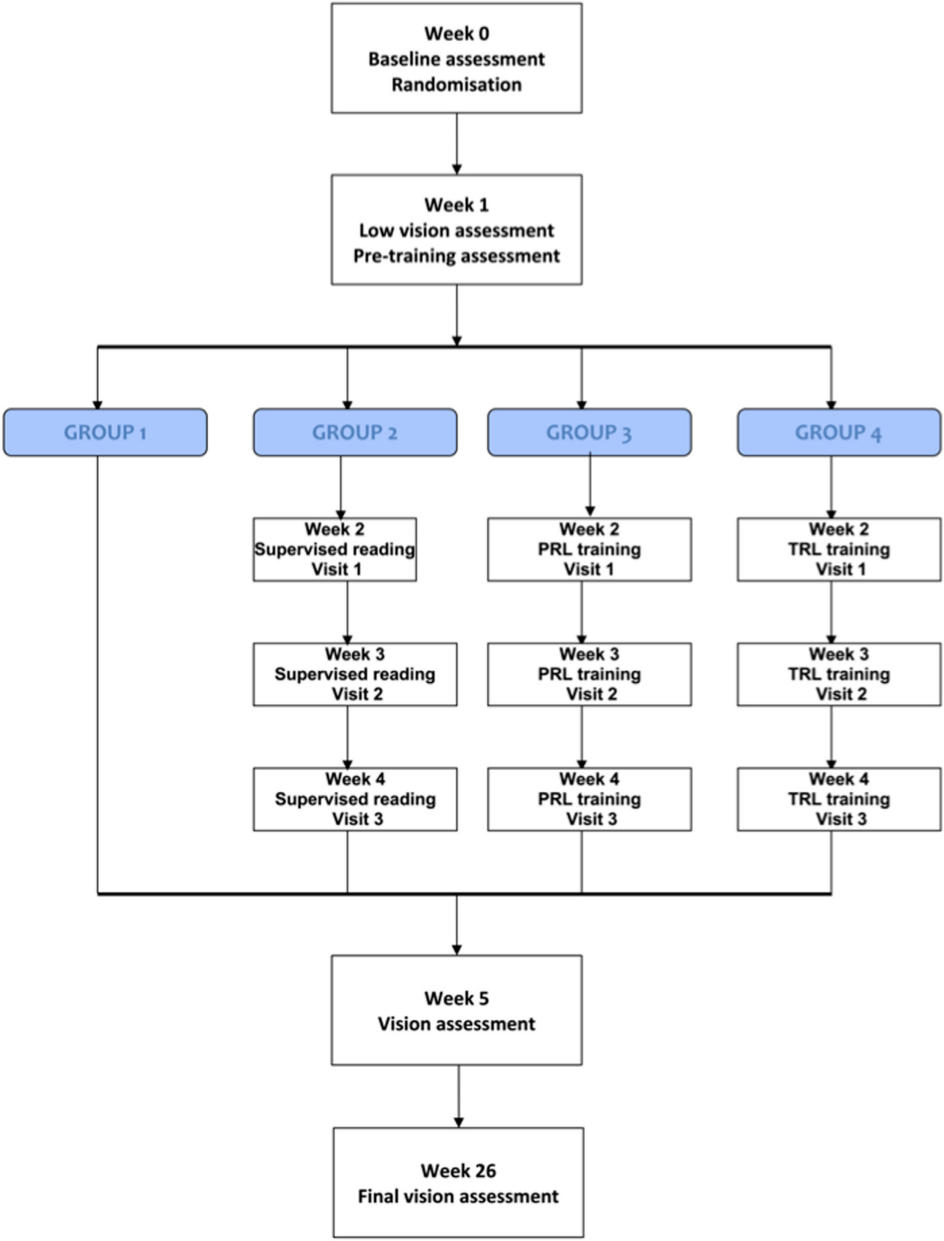
Flowchart of study visits for each group. PRL = preferred retinal locus; TRL = trained retinal locus.

Following baseline assessment, participants were randomized into 4 groups (Figure 1).

Randomization was performed using minimization to balance groups for type of AMD (dry/wet); AMD treatment (treated/untreated); visual acuity (0.5 logMAR to 0.8 logMAR/> 0.8 logMAR); and age (< 80 years/> 79 years).

All treatment and training was performed by a qualified optometrist with experience in hospital low vision clinics and additional instruction in eccentric viewing training.

### *Group 1:Control Group*

This group received standard low vision rehabilitation only. This consisted of refraction, prescription of spectacles, demonstration and supply of optical low-vision aids, demonstration of electronic low vision aids and, where appropriate, referral to local sensory teams (rehabilitation services provided by local councils), counselling services, mobility training, falls teams, or an eye clinic liaison officer (who links the patient to appropriate local charity and state services). Optical low-vision aids, including illuminated and nonilluminated hand and stand magnifiers, high addition reading glasses, spectacle mounted distance and near telescopes, and hand-held monocular telescopes and binoculars, were supplied at no cost to the participant.

### *Group 2:Control Group Plus Supervised Reading*

This group received low vision rehabilitation plus three 45-minute sessions of supervised reading. Participants chose from a selection of books and magazines of varying print sizes or used their own reading material. Participants sat in a quiet room with an investigator and were asked to read silently, using any prescribed spectacles or low vision aids. They were allowed to take breaks during the session.

### *Group 3:Eccentric Viewing Training at PRL*

This group received standard low-vision rehabilitation plus three 45-minute sessions of PRL training. Full details of the training are given in the Supplementary Material. Briefly, training was performed on the eye with better visual acuity, with the other eye patched. An Amsler chart with a superimposed cross (with lines linking the top left and bottom right corner, and the bottom left and top right corners) was used to determine PRL position. Participants were asked to use this chart to determine the clearest area of their visual field (which was expected to be the center of the chart if they were already using an optimal PRL, but a noncentral region of the chart if they were not using the best PRL). The PRL position was explained and demonstrated on various tasks, including a clock and a reading task. Participants were asked to make eye movements to this PRL. Initial training was performed without magnifiers, with low vision aids introduced once the concept was understood.

### *Group 4:Eccentric Viewing Training at TRL*

This group received standard low-vision rehabilitation plus three 45-minute sessions of TRL training. Full details of the training are given in the Supplementary Material. Briefly, training was performed on the better eye, with the other eye patched. A biofeedback technique was performed on a microperimeter (MP-1, Nidek Instrumants) to train participants to fixate with a location in the superior retina (inferior visual field) for periods of 15 seconds at a time. The biofeedback session lasted 15 minutes. After this, eye movement training to the TRL was performed as in Group 3.

#### Homework

Participants in groups 3 and 4 were asked to practice these techniques 4 to 5 times daily between visits. At each subsequent visit, a verbal check was made to ensure that the homework exercises were performed.

#### Outcome Measures

Outcome measures were recorded by a research assistant who was masked to group allocation.

#### Visual Measures

Visual acuity was measured monocularly using a 4-m logMAR chart. Contrast sensitivity was measured monocularly using a Mars chart (Mars Perceptrix) at 50 cm. Appropriate refractive correction was worn for all tests.

#### Primary Outcome Measure: Patient-Reported Visual Task Ability

The primary outcome measure for this study was visual task ability assessed using the AI after 6 months. The AI is a validated visual function questionnaire where people are asked to self-report their ability to perform 50 different goals (for example: using public transport, watching television, and communicating with people face to face) which are grouped into 3 objectives: daily living, social interactions, and recreation.^38^ The full version of the AI has 459 individual tasks as part of these 50 goals, but for this study the AI performed at goal level only. For each goal, participants were asked how important each goal was to them and asked to respond "not," "somewhat," "moderately," or "very" important. If their response was anything other than "not important," participants were asked how difficult they found it to achieve the goal: "not," "somewhat," "moderately," or "very" difficult, or "impossible."

Results from the AI were expressed in logits, which is the inverse of the standard logistic function of the model created by the responses to the questionnaire.^45^ There is a linear relationship between the logit value and vision utility,^46^ with higher (more positive) values reflecting better self-reported visual task ability.

The AI has been shown to have strong validity and reliability in measuring visual task ability^36^, ^37^, ^38^^,^^47^ and to be responsive to the effects of low vision rehabilitation.^48^

#### Secondary Outcome Measure: Reading Performance

Secondary outcome measures included multiple aspects of reading performance, with and without low vision aids. Maximum reading speed, critical print size, and reading acuity were assessed using the MNREAD test (Regents of the University of Minnesota) was performed with both eyes open. The chart was held 25 cm from the eye for people with distance visual acuity worse than 6/36 and 40 cm away for those with better vision. Appropriate refractive correction was worn.

Participants were advised to read each sentence as quickly and accurately as possible and were aware they were being timed. Each sentence was revealed in turn and the time between the sentence being revealed and the final word being read was measured. Errors were recorded and testing continued until no words of a sentence were read or after 30 seconds passed with no words being read.

Maximum reading speed was recorded for the fastest read sentence. Critical print size was calculated as the smallest print size which was read within 10% of the mean time of the 3 fastest read sentences. Reading acuity (RA) was calculated as:

RA=K−(sentencesattempted×0.1)+(errors×0.01)

K was 1.4 if the test was performed at 40 cm and 1.6 when the test was at 25 cm.

Magnifier assisted reading speed was measured using the International Reading Speed Test^49^ at 2 print sizes: 9 point Arial (approximately equivalent to N5 or 0.625M) and 18 point Arial (about N10 or 1.25M). Participants were allowed to place the test wherever they wished and could use their own optical, but not electronic, low vision aids. After 2 practice paragraphs, a new paragraph was selected at random and participants were asked to read it aloud. The time between the sentence being revealed and the last word being read was measured. If the paragraph was not read completely within 90 seconds, the test was stopped and the number of words read in 90 seconds was recorded. The same test was not used on consecutive visits.

Reading comprehension was assessed using the Morgan Low Vision Reading Comprehension Assessment test (Fork in the Road Vision Rehabilitation Services).^50^ The investigator and participant selected print of a comfortable font size. Participants positioned the print themselves and adjusted the task lighting. They could use their own optical magnifiers (but not electronic magnifiers). Participants were asked to read each sentence once, silently, and to select an appropriate word which could fit in the gap in the sentence (for example, in the sentence “An athlete runs faster _______ than indoors,” “outdoors” would be an acceptable choice but "fish" would not). After 3 practice sentences, testing began with the first test sentence and continued until the test has been completed or the reader made 4 consecutive errors.

The total number of correct answers gave the raw Morgan comprehension score. An approximate grade level equivalent (GLE) was calculated using the formula:

GLE=(1.266×RawScore)−5.066

#### Secondary Outcome Measure: Fixation Stability

A further secondary outcome measure was fixation stability was measured using a Nidek MP-1 microperimeter (Nidek Instruments). Both pupils were dilated and participants were dark-adapted for 10 minutes before fixation was tested. Eye position was recorded for 30 seconds as participants observed a red 2° fixation cross. Participants were instructed to look directly at the cross. The area of a bivariate contour ellipse encompassing 68% of fixation positions was recorded.

#### Sample Size

Sample size was calculated for the primary outcome measure of task ability measured using the AI, using Power and Sample size calculation (PS) for Windows (version 3.0, Vanderbilt University), with type I error rate (α) of 0.05 (2-tailed); standard deviation (SD) of AI scores of 0.97 logit (based on previous work from our group^40^); and a minimum important difference of 0.7 logit (equivalent to a 0.5 logMAR [5-line] change in visual acuity). This power calculation showed 41 participants were required per group. To allow for a dropout rate of 20%, 50 participants were recruited to each group.

#### Statistical Method

Analysis was performed on an intention to treat basis.^51^ For participants who received training but were lost to follow-up, visual acuity data were imputed from the last measured visual acuity at a training visit. Analysis of variance (ANOVA) was used to identify differences between groups with appropriate posthoc tests used in the case of ANOVA returning a significant result. The planned analyses were published prior to data collection on clinicaltrials.gov (NCT01499628). Analyses were performed in JMP Pro (version 17.0.0, JMP Statistical Discovery LLC) and MATLAB (Mathworks Inc).

#### Ethics

Ethical approval was obtained from the Cambridge 3 Research Ethics Committee. All participants gave informed consent prior to data collection. The study conformed to the tenets of the Declaration of Helsinki. The study was registered with clinicaltrials.gov (NCT01499628).

## Results

### Patient Characteristics

A total of 2616 patients were screened, of whom 682 met the inclusion criteria. Two hundred participants were recruited. Baseline visits took place between August 10, 2011 and December 20, 2012.

**Figure 2:**
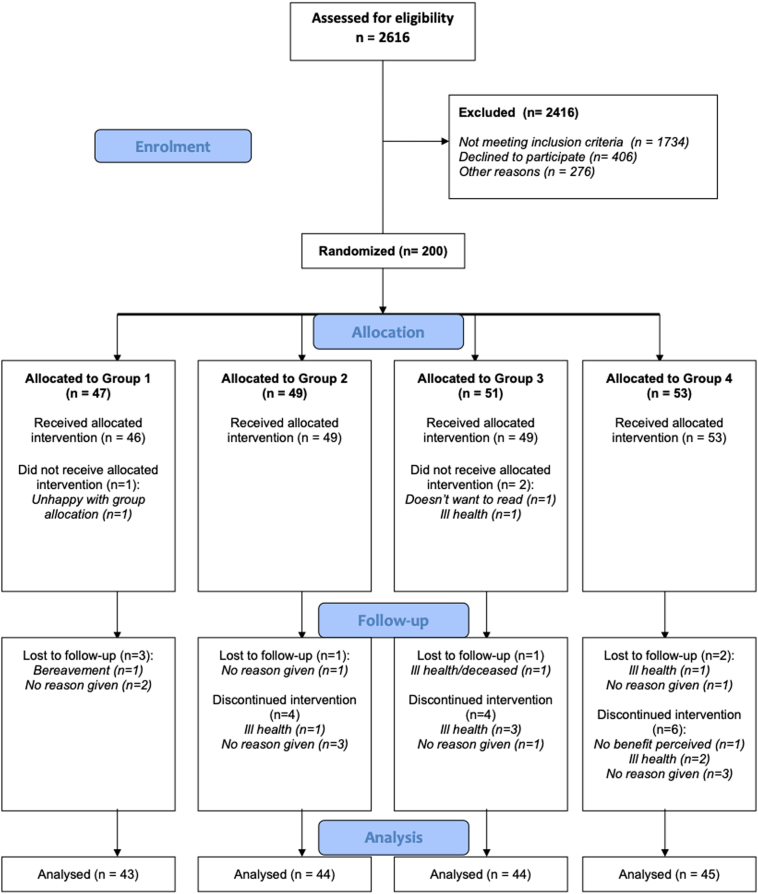
CONSORT flowchart for the study.

One participant in Group 1 (control) and 2 in Group 3 (PRL training) declined to take part after randomization. Twenty-one did not complete follow-up (3 in Group 1, 5 in Group 2, 5 in Group 3, and 8 in Group 4 [Figure 2]).

Baseline characteristics for each group are shown in Table 1 and changes in outcome measures are shown in Table 2, Table 3, Table 4.

**Table 1:** Baseline Characteristics of Participants in Each Group

Group	Treatment	Age (Years)	% Female	% Dry AMD	Visual Acuity (logMAR)	Contrast Sensitivity (Log Units)
1	Control	81.6 (7.4)	57	32	0.62 (0.18)	1.07 (0.32)
2	Supervised reading	82.4 (8.4)	61	35	0.74 (0.27)	1.10 (0.27)
3	PRL training	80.3 (6.0)	55	34	0.68 (0.25)	1.16 (0.27)
4	TRL training	81.4 (6.7)	70	32	0.64 (0.22)	1.13 (0.28)

AMD = age-related macular degeneration; logMAR = logarithm of the minimum angle of resolution; PRL = preferred retinal locus; TRL = trained retinal locus.

All columns show mean (standard deviation) values.

**Table 2:** Mean and SD of Visual Task Ability Pre- and Posttreatment for Each Group

Group	Treatment	Visual Task Ability (Logit)					
		Pretreatment		Posttreatment		Difference Pre- to Post-treatment	
		Mean	SD	Mean	SD	Difference (95% CI)	ANOVA
1	Control	−0.0358	0.983	−0.181	1.00	−0.145 (−0.295 to 0.00461)	*F*_(3,174)_ = 1.48 *P* = 0.22
2	Supervised reading	0.129	0.899	0.149	1.04	0.0198 (−0.143 to 0.183)	
3	PRL training	0.0421	0.843	−0.153	0.916	−0.195 (−0.3616 to −0.0282)	
4	TRL training	0.0284	0.956	−0.0109	0.930	−0.0393 (−0.210 to 0.131)	

ANOVA = analysis of variance; CI = confidence interval; PRL = preferred retinal locus; SD = standard deviation; TRL = trained retinal locus.

**Table 3:** Mean and SD of Reading Outcomes Pre- and Posttreatment for Each Group

Outcome	Group	Treatment	Pretreatment		Posttreatment		Difference Pre-Post Treatment	
			Mean	SD	Mean	SD	Difference (95% CI)	ANOVA
Max reading speed (wpm)	1	Control	104	69	82.8	59	−21.2 (−36 to −6.6)	*F*_(3,168)_ = 1.17 *P* = 0.32
	2	Supervised reading	105	57	93.0	48	−12.0 (−22 to −1.6)	
	3	PRL training	108	59	87.6	49	−20.7 (−30 to −11)	
	4	TRL training	100	59	72.9	47	−26.7 (−38 to −15)	
Critical print size (logMAR)	1	Control	1.21	0.22	1.20	0.28	−0.0088 (−0.060 to 0.042)	*F*_(3,170)_ = 0.97 *P* = 0.41
	2	Supervised reading	1.16	0.23	1.20	0.21	0.0267 (−0.042 to 0.096)	
	3	PRL training	1.12	0.26	1.17	0.21	0.0452 (−0.012 to 0.10)	
	4	TRL training	1.15	0.25	1.11	0.26	−0.045 (−0.091 to 0.00019)	
Reading acuity (logMAR)	1	Control	0.864	0.34	0.951	0.33	−0.0878 (−0.15 to −0.025)	*F*_(3,170)_ = 0.91 *P* = 0.44
	2	Supervised reading	0.800	0.33	0.874	0.34	−0.066 (−0.14 to 0.0088)	
	3	PRL training	0.804	0.31	0.851	0.31	−0.0471 (−0.10 to 0.066)	
	4	TRL training	0.827	0.34	0.928	0.35	−0.10 (−0.16 to −0.043)	
Magnifier reading speed (small print, wpm)	1	Control	55.4	43	65.6	40	10.2 (−0.56 to 21)	*F*_(3,147)_ = 0.57 *P* = 0.64
	2	Supervised reading	51.0	38	63.3	34	12.3 (0.32 to 24)	
	3	PRL training	57.7	36	61.6	34	3.82 (−2.4 to 10)	
	4	TRL training	51.3	45	61.3	40	10.1 (−0.18 to 20)	
Magnifier reading speed (large print, wpm)	1	Control	68.2	48	69.6	40	1.36 (−7.2 to 9.9)	*F*_(3,165)_ = 0.22 *P* = 0.88
	2	Supervised reading	64.1	39	64.8	36	0.69 (−7.6 to 9.0)	
	3	PRL training	74.9	41	72.8	37	−2.10 (−11 to 6.4)	
	4	TRL training	59.4	41	61.7	40	2.25 (−4.4 to 8.9)	
Comprehension (Grade level equivalent)	1	Control	12.1	5.0	12.2	4.9	0.159 (−1.6 to 1.9)	*F*_(3,131)_ = 0.80 *P* = 0.50
	2	Supervised reading	11.2	4.1	10.7	4.0	−0.54 (−1.8 to 0.73)	
	3	PRL training	10.7	4.3	11.1	4.3	0.409 (−1.2 to 2.0)	
	4	TRL training	10.9	3.9	11.6	2.7	0.774 (−0.35 to 1.9)	

ANOVA = analysis of variance; CI = confidence interval; logMAR = logarithm of the minimum angle of resolution; PRL = preferred retinal locus; SD = standard deviation; TRL = trained retinal locus; wpm = words per minute.

**Table 4:** Mean and SD of Fixation Stability Values Pre- and Posttreatment for Each Group

Group	Treatment	Fixation Stability (Log Degrees^2^)					
		Pretreatment		Posttreatment		Difference Pre- to Posttreatment	
		Mean	SD	Mean	SD	Difference (95% CI)	ANOVA
1	Control	0.680	0.82	0.781	0.62	−0.10 (−0.35 to 0.15)	*F*_(3,137)_ = 0.57 *P* = 0.64
2	Supervised reading	0.66	0.47	0.81	0.50	−0.15 (−0.27 to −0.027)	
3	PRL training	0.704	0.57	0.730	0.56	−0.026 (−0.13 to 0.08)	
4	TRL training	0.681	0.57	0.792	0.61	−0.11 (−0.22 to 0.00069)	

ANOVA = analysis of variance; CI = confidence interval; PRL = preferred retinal locus; SD = standard deviation; TRL = trained retinal locus.

### Change in Vision over the Study

On average, visual acuity deteriorated by 0.20 logMAR (SD: 0.47) over the study, from a mean value of 0.67 logMAR (SD: 0.24) to 0.87 logMAR (SD: 0.41). There was no significant difference in visual acuity change between those with dry AMD (mean change = 0.21 logMAR [SD: 0.42]) and those with wet AMD (mean change = 0.19 logMAR [SD: 0.50], ANOVA between wet and dry AMD, *F*_(1,166)_ = 0.08, *P* = 0.78). There was no difference between groups in the magnitude of visual acuity change (ANOVA between groups, *F*_(3,166)_ = 0.75, *P* = 0.75).

On average, contrast sensitivity deteriorated by 0.18 log units (SD: 0.34), from a mean of 1.17 log dB (SD: 0.26) to 0.99 log dB (SD: 0.40). Change in contrast sensitivity was linearly related to change in visual acuity (Pearson *r* = −0.47, *P* < 0.001). There was no significant difference in contrast sensitivity change between those with dry AMD (mean change = −0.15 logMAR [SD: 0.34]) and those with wet AMD (mean change = −0.19 logMAR [SD: 0.35], ANOVA between wet and dry AMD, *F*_(1,166)_ = 0.57, *P* = 0.45). There was no difference between groups in the magnitude of contrast sensitivity change (ANOVA between groups, *F*_(3,166)_ = 2.27, *P* = 0.08).

### Primary Outcome Measure: Patient-Reported Visual Task Ability

**Figure 3:**
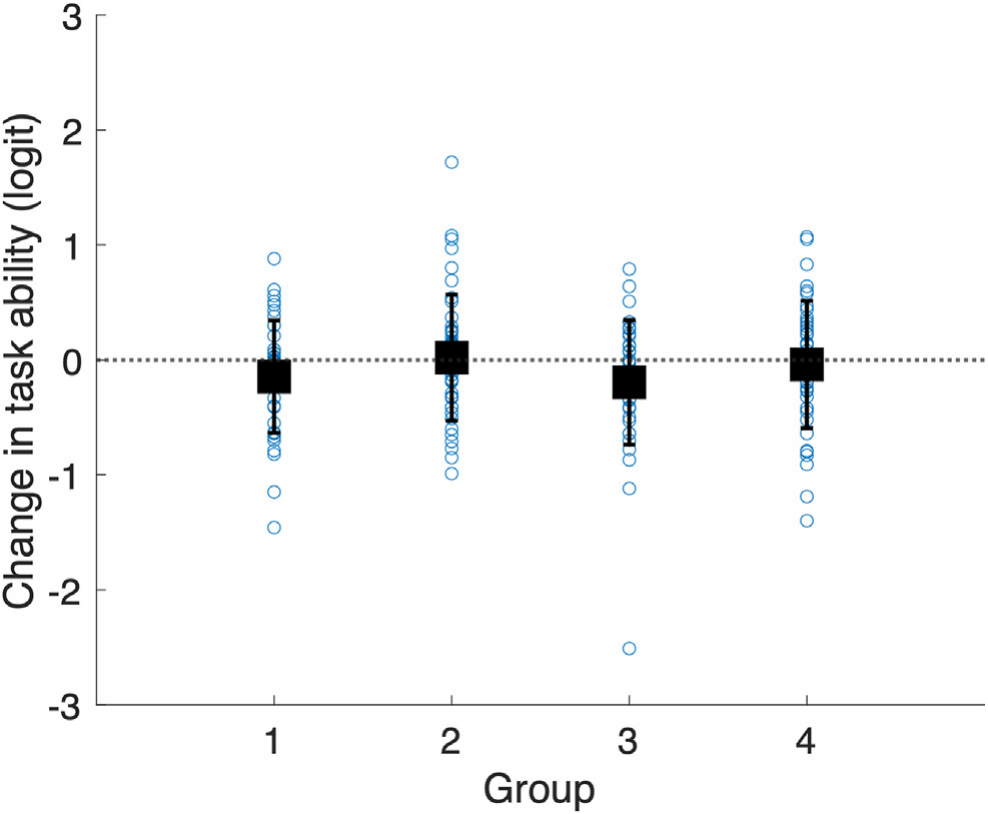
Change in task ability by group. Blue circles show individual participants. Black squares show mean value. Error bars show 1 standard deviation. Dashed line shows no change.

There was no significant difference between groups in the change in task ability (ANOVA between groups, *F*_(3,174)_ = 1.48, *P* = 0.22) (Table 2, Figure 3).

There was a linear relationship between visual acuity and visual task ability at baseline, with poorer visual acuity being associated with poorer task performance (Pearson *r* = −0.372, *P* < 0.001). However, change in visual acuity was not related to change in task performance (*r* = −0.102, *P* = 0.22).

### Secondary Outcome Measure: Reading Performance

**Figure 4:**
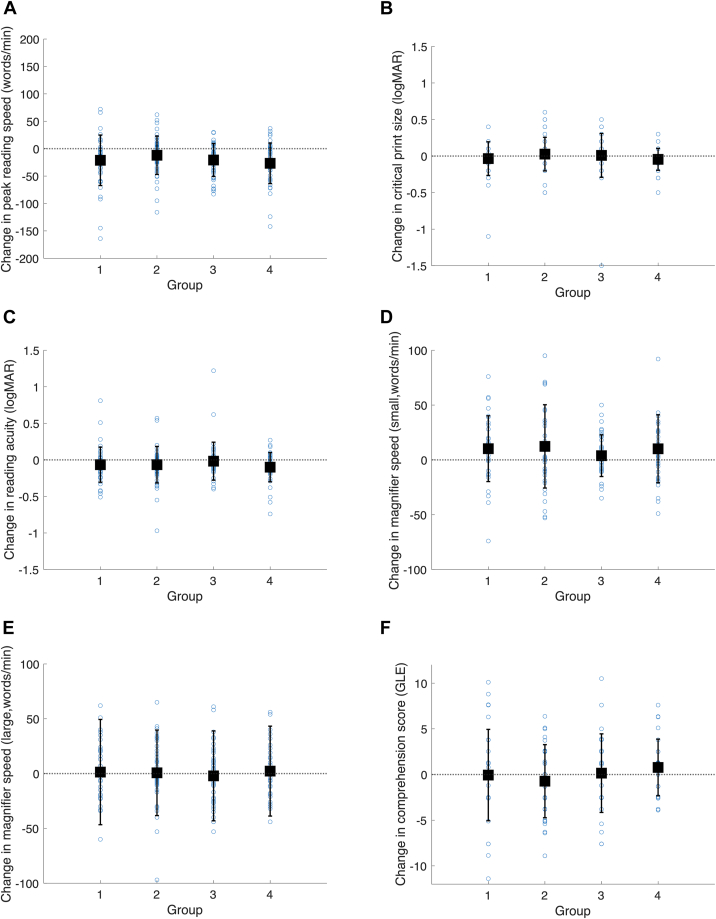
Change in reading outcome measures by group. **A,** Peak reading speed. **B,** Critical print size. **C,** Reading acuity. **D,** Magnifier aided reading speed (small print). **E,** Magnifier aided reading speed (large print). **F,** Comprehension score. GLE = grade level equivalent; logMAR = logarithm of the minimum angle of resolution.

All reading data are shown in Table 3 and Figure 4. There was a significant drop in maximum reading speed over the study, from a mean value of 104 words/minute to a mean value of 84 words/minute (matched pairs, *t*_(171)_ = −7.04, *P* < 0.001). There was no significant difference in reading speed change between groups (ANOVA between groups, *F*_(3,168)_ = 1.17, *P* = 0.32).

There was also a significant drop in reading acuity (from 0.83 to 0.89 logMAR, matched pairs, *t*_(173)_ = 3.52, *P* < 0.001). There was no significant difference in reading acuity change between groups (ANOVA between groups, *F*_(3,170)_ = 0.91, *P* = 0.44).

There were no statistically significant differences between groups in the change in critical print size (ANOVA between groups, *F*_(3,170)_ = 0.97, *P* = 0.41), magnifier-assisted reading speed for small print (ANOVA between groups, *F*_(3,147)_ = 0.57, *P* = 0.64), magnifier-assisted reading speed for large print (ANOVA between groups, *F*_(3,165)_ = 0.22, *P* = 0.88), or reading comprehension (ANOVA between groups, *F*_(3,131)_ = 0.80, *P* = 0.50); (Table 3, Figure 4).

### Secondary Outcome Measure: Fixation Stability

**Figure 5:**
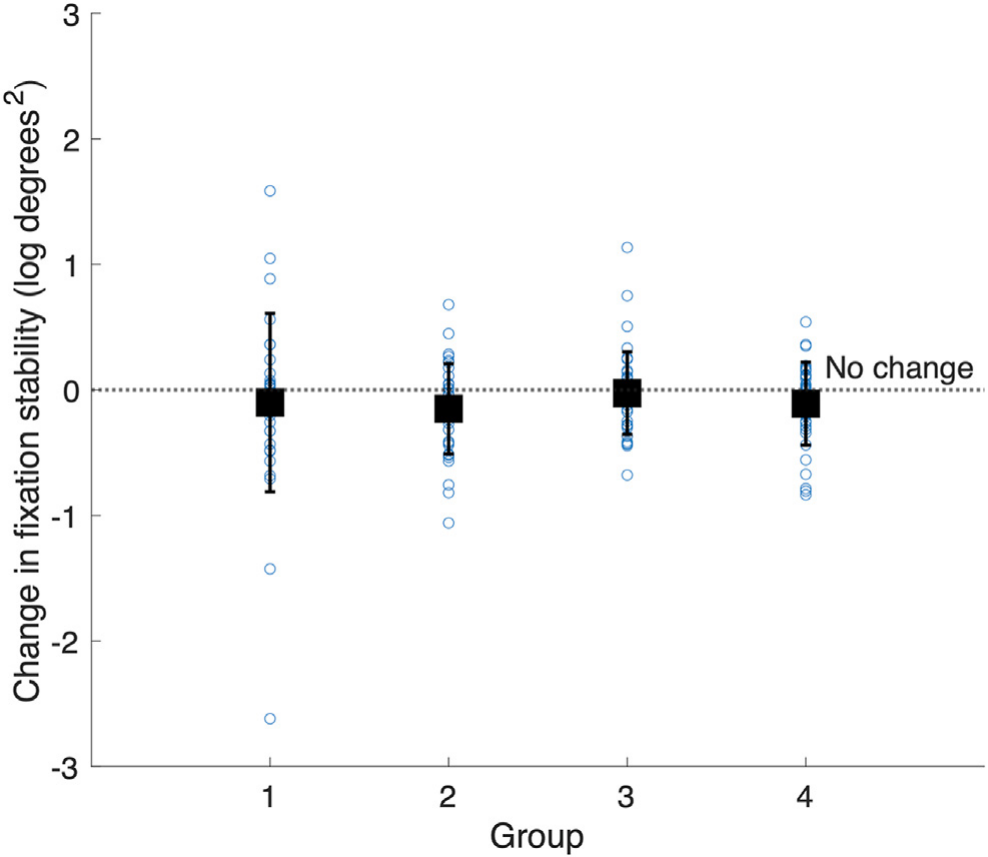
Change in fixation stability by group. Positive values represent deteriorating fixation stability. Error bars show 1 standard deviation.

On average, fixation stability deteriorated by 0.10 log degrees^2^ (SD: 0.44), from a mean value of 0.67 log degrees^2^ (SD: 0.59) to 0.78 log degrees^2^ (SD: 0.57) (matched pairs: *t*_(144)_ = 2.59, *P* < 0.01). There was no significant difference between groups in the change in fixation stability over the study (ANOVA between groups, *F*_(3,137)_ = 0.57, *P* = 0.64, Table 4, Figure 5).

There was no significant difference in fixation stability change between those with dry AMD (mean deterioration = 0.15 log degrees^2^ [SD: 0.53]) and those with wet AMD (mean deterioration = 0.06 log degrees^2^ [SD: 0.40], ANOVA between wet and dry AMD, *F*_(1,142)_ = 1.17, *P* = 0.28).

## Discussion

We found no significant effect of eccentric viewing training on task ability, reading performance, or fixation stability. Visual acuity and contrast sensitivity deteriorated in every group, reflecting the difficulty of rehabilitation in a group of people with an inherently progressive disease.

Unlike many previous studies of low vision rehabilitation,^32^ we included 2 large control groups, but did not find the increased contact time for the supervised reading control group (Group 2) led to better outcomes than the control group with no additional visits (Group 1).

It is remarkable how little of a placebo effect our participants experienced. All were aware that there were control and intervention arms in the study and it was obvious to them if they were receiving training. We did not expect visual acuity or fixation characteristics to be affected by a placebo, but anticipated that the contact time might improve self-reported task ability.

It is not clear why our subjects did not show the improvement in performance found by, for example, Nilsson^23^ or Watson.^25^ We consider 4 possible explanations for this: differences in the amount of training received, in the experience of those performing the training, in the type of training received, and in the patient population studied.

Our participants received three 45-minute training sessions. There is wide variability in the amount of training suggested by other authors, ranging from eight 10-minute sessions^31^ to 30 to 60 hours over up to 6 weeks.^26^ More training may have lead to more impressive outcomes in this study, but the lack of any effect after 135 minutes of training does not suggest this.

Twenty-four of our 200 participants did not complete all the training visits. Their data are included in our results, as we performed an intention to treat analysis where all participants were included, even if they did not complete the training procedure. We are aware that this analysis can underestimate clinical effectiveness.^51^

Our eccentric viewing training was performed by experienced low-vision optometrists, who received additional instruction from a very experienced eccentric viewing trainer. However, they did not have direct clinical experience of this technique before this study. More experience of eccentric viewing training may lead to better outcomes.

Each participant in our intervention groups received the same type and amount of eccentric viewing training. In clinical practice, eccentric viewing training may be tailored to the individual, maximising the chance of success. It may be that some eccentric viewing trainers would elect to train only those most likely to benefit from training.

Our participants had a broad range of visual acuity, including many with better acuity than those trained by Nilsson and others. Daibert-Nido and colleagues recently showed visual acuity benefits from biofeedback training only for those with visual acuity of 0.8 logMAR or poorer.^52^ It is possible that if we had stricter inclusion criteria, we may have identified more benefit from eccentric viewing training.

Some of our participants had good visual acuity but some regions of absolute scotoma on microperimetry, suggesting that they had paracentral scotomas. Eccentric viewing training is challenging in this population, particularly in those with ring scotomas.^53^ Paracentral “horseshoe” and “ring” scotomas are often observed in people with dry AMD.^54^ Calabrèse et al^55^ have shown that people with wet AMD read more quickly than those with dry AMD and speculated that training may be more advantageous for those with dry AMD. We also found that people with wet AMD read faster at baseline (MNREAD peak reading speed at baseline: dry AMD: 93 words/minute; wet AMD: 110 words/minute [1-tailed *t*-test between wet and dry AMD, *t*_(1,168)_ = 1.72, *P* < 0.05]), and it may be that training would be more effective for this subgroup of people with AMD.

Despite the lack of a group effect, improvements were seen in task ability for some people who received training, of up to 1.7 logit. Of the 10 participants with the largest improvement in task ability, 1 was in the control group, 1 was in the PRL training group, 4 were in the supervised reading group, and 4 received TRL training. Does this mean that eccentric vieiwng training should be offered despite the lack of effect in our study? We would argue that any training should not be offered routinely to people with progressive macular disease, but can not rule out the potential benefit of training for some people, particularly those in the final stages of the disease. Future research should identify those most likely to benefit from this intervention.

### Limitations

Including participants with a wide range of visual acuity and with diverse forms of age-related macular disease may have reduced the size of any treatment effect in this study. Tighter inclusion criteria (perhaps of only those with more severe vision impairment and without paracentral scotoma) may have demonstrated a larger effect of eccentric viewing training.

We had a large refusal rate; twice as many people declined to participate in the study than agreed to take part. This may reflect the time burden of travelling into a city center hospital location for training. It is possible that those with most need of training may have been those who felt least able to attend several study visits. Offering eccentric viewing training in people’s homes, in local health facilities, or online may increase access to these services.

We did not ask participants in Groups 3 or 4 to keep a diary of their homework exercises, instead relying on a verbal check at each research visit. It is possible that some participants may not have performed these exercises, particularly if they did not perceive any immediate effect from the training program. In retrospect, it may have been wise to measure their adherence to the protocol.

## Conclusion

In our large, carefully controlled, randomized clinical trial we found no effect of eccentric viewing training on task ability, reading performance, or fixation stability. On average, visual acuity worsened in all groups. This suggests that disease progression may outweigh any benefits of training. Our results do not support the routine provision of eccentric viewing training for people with progressing age-related macular disease.
