# Supplementary material for: Eccentric Viewing Training for Age-Related Macular Disease: Results of a Randomized Controlled Trial (the EFFECT Study)
Source: Ophthalmol Sci. 2023 Oct 31;4(2):100422. doi: 10.1016/j.xops.2023.100422 (PMC10767206; doi:10.1016/j.xops.2023.100422)
Supplement: Appendix S1 [file mmc1.pdf]

Standard Operating Procedure

Title: **Eccentric Viewing Training at the PRL**

1. Purpose

To provide guidance and a standard procedure for performing eccentric viewing training at the Preferred Retinal Locus (PRL).

2. Scope

This standard operating procedure (SOP) will be used when performing eccentric viewing training at the PRL to ensure that all procedures are performed consistently. Adherence to this SOP will also ensure the proper treatment of participants and that all data are available for future analysis.

3. Area of application

WAU

4. Personnel

All research personnel

5. Equipment

Optima Low Vision Eccentric Viewing Reading Cards (Section 1 & 2)

Magnifiers

Amsler chart

Tape measure

Adjustable chart stand

Softalind alcohol hand rub

Trial frame

Precision vision illumination box and stand

ETDRS acuity charts

Clipboard

Trial lenses

Lighting needs to be 450lux

Bailey Lovie near vision chart

6. Principle of the procedure

This procedure describes the method for performing eccentric viewing training at the PRL. It is designed to teach patients with Age Related Macular Degeneration to use a different (peripheral) area of the retina other than the fovea to perform tasks such as reading. This training is performed at the PRL (preferred retinal locus), which is an area on the retina that the patient 'uses' when trying to fixate eccentrically to the fovea. It is designed to improve stability and make it easier/quicker to for example, read.

7. Assessment of health risk

Care is taken to avoid skin cross contamination: the researcher should clean their hands before and after contact with each participant using alcohol solution.

8. Method

8.1 Participant History

a. Explain the cause of visual loss to the participant
b. Explain why it only affects the central vision and how this causes everyday tasks e.g. reading to become difficult
c. Explain how steady eye strategy works
d. Ask participant their working distance for reading/other tasks
e. Note if they have a hand tremor/disability

8.2 Practical

a. Record R & L distance visual acuity (monocular) (see Visual Acuity SOP)
b. Repeat 8.2a, but advise the participant that they can move their head about to get better vision. Note down the direction they are looking.
c. Patch the eye with the worst acuity and continue all further tests with the better acuity eye
d. Record near VA with the Optima Near vision magnification prediction chart on a clip board with the participant sitting in a comfortable position. Start with the largest size print and work down the card until the participant struggles to read. They can use appropriate near vision spectacle correction.
e. Repeat 8.2d, but ask the participant to keep the card still and move their eyes around to improve their near vision acuity. Note down the direction they are looking in order to get an improved near vision reading.
f. Using Chart 1 (Amsler), ask the participant to look at the centre of the cross and move their eyes so it looks blurry.
g. Ask the participant where the squares are clearest up or down and then right or left and note this down.

h. Using Chart 1 (Amsler) hold a pen in the opposite direction to where the participant reported the squares were clear in 8.2g EXAMPLE: squares clearest right & below, so hold pen left & up i.e. their PRL
i. Ask the participant to look at the pen until misty and simultaneously, see if the cross is now clear. Explain why this works.
j. Ask participant to look away and repeat 8.2i several times until automatic
k. Repeat 8.2h-8.2j, but without a pen
l. Demonstrate the PRL using a clock face
m. Place your hand in the PRL area and ask the participant to look at your hand until it goes misty, but observe your face simultaneously. Demonstrate that by looking at your hand (i.e. PRL) whilst observing features on your face is clearer than looking at your face directly. Once the participant appreciates this, see if they can do it with the hand as a guide.
n. Using the Optima Near Vision magnification predication chart, place a pen in the participant's PRL and ask the participant to read the print until it is too small to see. Also, point out that it is best to move the clipboard but not their eyes. Repeat this but without the aid of the pen.
o. Once the participant is confident with the technique, re-evaluate their current magnifiers and examine whether it is possible to reduce magnification now that the participant is using the PRL to read. Keep reminding the participant not to move their eye/head/magnifier only slide the book.
p. Repeat near vision acuity with the Bailey Lovie chart
q. Repeat distance visual acuity but using the PRL
r. Repeat distance visual acuity but using the PRL
s. Using the Functional Field Test Chart, ask the participant to read the sentences keeping their eyes in a position where the letters are clearest (i.e. using the PRL). Observe the participant and note which sentence they make their first eye movement including the number of words.
t. Using Chart 4, choose a size that's similar to near acuity
u. Ask the participant to look at number 5 & simultaneously see if they can see numbers 4 & 6, then 3 & 7, then 2 & 8.
v. Advise participant to practice little and often – 4x-5x a day looking at bills/letters for about 10mins, as well as completing the homework give from the training
w. Arrange a follow-up appointment for one weeks' time to a total of three visits

8.3 <u>Close down</u>

a. Store all of the equipment.
