# Supplementary material for: Eccentric Viewing Training for Age-Related Macular Disease: Results of a Randomized Controlled Trial (the EFFECT Study)
Source: Ophthalmol Sci. 2023 Oct 31;4(2):100422. doi: 10.1016/j.xops.2023.100422 (PMC10767206; doi:10.1016/j.xops.2023.100422)
Supplement: Appendix S2 [file mmc2.pdf]

# Standard Operating Procedure

Title: **Eccentric Viewing Training at the Trained Retinal Locus (TRL)**

1. **Purpose**

   To provide guidance and a standard procedure for performing eccentric viewing training at the Trained Retinal Locus (TRL).

2. **Scope**

   This standard operating procedure (SOP) will be used when performing eccentric viewing training at the TRL to ensure that all procedures are performed consistently. Adherence to this SOP will also ensure the proper treatment of participants and that all data are available for future analysis.

3. **Area of application**

   WAU

4. **Personnel**

   All research personnel

5. **Equipment**

   Nidek Micro Perimeter MP-1

   Disposable eye patch

   Micropore tape

   Softalind alcohol hand rub

   Alcotip swabs

   Optima Low Vision Eccentric Viewing Reading Cards (Section 2)

   Magnifiers

   Amsler chart

   Tape measure

   Adjustable chart stand

   Trial frame

Precision vision illumination box and stand

ETDRS acuity charts

Clipboard

Trial lenses

Lighting needs to be 450lux

Bailey Lovie near vision chart

## 6. Principle of the procedure

This procedure describes the method for performing eccentric viewing training at the TRL. It is designed to teach patients with Age Related Macular Degeneration to use the upper part of the retina (TRL) to perform tasks such as reading. This training is performed using the biofeedback feature on the MP-1. This training is designed to improve stability and make it easier/quicker to for example, read.

## 7. Assessment of health risk

Care is taken to avoid skin cross contamination: the researcher should clean their hands before and after contact with each participant using alcohol solution.

## 8. Method

### 8.1 Participant History

a. Explain the cause of visual loss to the participant
b. Explain why it only affects the central vision and how this causes everyday tasks e.g. reading to become difficult
c. Ask participant their working distance for reading/other tasks
d. Note if they have a hand tremor/disability

### 8.2 Setting up the MP-1 & preparing the patient

a. Remove the protective cover and the lens cap. Switch on the MP-1 and computer and allow 10 minutes before starting an exam so that background and stimuli luminescence can stabilise.
b. Clean the response button, forehead and chin rests using ethanol wipes
c. Dark adapt the participant for 5 minutes in the examination room with the lights switched off and the doors closed.
d. Seat the participant in front of the MP-1 in a comfortable relaxed position.
e. Hand the participant an occluder and ask them to cover the eye with the worst acuity.
f. Ask the participant to sit forward and place their head on the chin rest (on the left hand side if testing the right eye and vice versa) and press their forehead firmly against the forehead rest.
g. Adjust the height of the chin rest using the wheel so that the eyes are level with the markings on the MP-1. Adjust the height of the table using the button underneath the table to ensure the participant is comfortable.
h. Log onto the Navis software

i. Select ‘MP1383 user’ and then microperimetry

j. Select participant from the list or create a new patient and enter details

k. Switch off the room lights as the exam will be conducted in darkness. Rotate the operator’s screen away from the participant to minimise the reflection of stray light towards the participant.

l. Select ‘*New Exam*’.

m. Click on ‘selected test’ from the examination toolbar on the bottom of the screen and choose ’10-2’

n. Click on‘Fixation’ from the examination toolbar on the bottom of the screen. Ensure that the appropriate target size is selected (2° cross). If the participant cannot see the cross at 2°, increase the size in 1° increments up to 4° until seen. The thickness of the cross in pixels should match the size in degrees (See section 8.1 for instructions on changing the size of the cross).

8.3 Acquiring a retinal image & Fixation

a. Ask the participant to look at the fixation cross
b. Move the MP-1 to the backmost position with the use of the joystick. Centre the participant’s eye on screen, lining it up with the markings by turning the joystick.
c. Once the pupil has been framed, move the joystick forward, gradually adjusting up/down and right/left alignment until focal distance (50 mm) is reached and the retina including part of the optic disc has been framed. Correct focal distance is indicated when two white spots overlaid on fundus image are in sharp focus and aligned with blue markers to left and right.
d. Adjust IR power to create slight “bloom” (overexposure that appears as white patch in centre) if necessary. (NB: If the participant is wearing contact lenses this may cause a reflection, but this should not interfere with testing.)
e. Input the participant’s spherical error at the bottom of the screen
f. Select ‘Fixation’
g. Remind the participant to look directly at the cross for the duration of the exam and ensure they are holding the response button.
h. When the image on screen is focused and stable press the button on the top of the joystick, or right click the mouse, with the curser on the image to get a still image on the screen. If the image presented is blurred press ‘*Stop*’ (from the examination toolbar underneath the large image), repeat section 8.4a to get the white dots central and in focus and begin fixation again until the image is clear. This is the image that will be displayed throughout the whole exam and the one that will be stored and used when presenting the results and printing.
i. Now a square will appear; using the mouse, position this over an area of high contrast (across the optic disc and a major vessel is recommended) until the high contrast features are illuminated in green and left click the mouse. This will start the exam.
j. The tracking window bar is illuminated in green when the retinal image is being tracked, and is red when tracking stops or when the exam is suspended. If tracking stops unexpectedly it may be necessary to reposition the participant or adjust the camera position.
k. Click the ‘*Stop*’ button after 30 seconds of tracked fixation.
l. Allow the participant to sit back and relax for a few moments before proceeding to the next test.

8.4 Biofeedback Training

a. Ask the participant to look at the fixation cross
b. Explain Biofeedback and why we are training a new retinal location to read
c. Move the MP-1 to the backmost position with the use of the joystick. Centre the participant's eye on screen, lining it up with the markings by turning the joystick.
d. Once the pupil has been framed, move the joystick forward, gradually adjusting up/down and right/left alignment until focal distance (50 mm) is reached and the retina including part of the optic disc has been framed. Correct focal distance is indicated when two white spots overlaid on fundus image are in sharp focus and aligned with blue markers to left and right.
e. Adjust IR power to create slight "bloom" (overexposure that appears as white patch in centre) if necessary. (NB: If the participant is wearing contact lenses this may cause a reflection, but this should not interfere with testing.)
f. Input the participant's spherical error at the bottom of the screen
g. Select 'Feedback'
h. Remind the participant to look directly at the cross for the duration of the exam and ensure they are holding the response button.
i. When the image on screen is focused and stable press the button on the top of the joystick, or right click the mouse, with the curser on the image to get a still image on the screen. If the image presented is blurred press '*Stop*' (from the examination toolbar underneath the large image), repeat section 8.4a to get the white dots central and in focus and begin fixation again until the image is clear. This is the image that will be displayed throughout the whole exam and the one that will be stored and used when presenting the results and printing.
j. Now a square will appear; using the mouse, position this over an area of high contrast (across the optic disc and a major vessel in the upper retina) until the high contrast features are illuminated in green and left click the mouse
k. Explain to the participant that in a moment they will need to look up from the fixation target and when the test start they will hear an intermittent sound which will increase in frequency the closer they get to fixating with the new retinal location (i.e. the closer the participant aligns the target retinal location with the fixation target).
l. Say that you will keep guiding them to move their eyes until they hear a continuous tone. One the tone is continuous, you will ask them to hold their position of gaze for 15 seconds.
m. The intermittent sound of the biofeedback procedure plus your guidance will help the participant align the selected retinal area with the fixation target.
n. Select an upper area of the retina to be trained. This should be at approximately the same radial distance from the old fovea (but not more than 10 deg) and in an area of 'optimum' sensitivity found by analysing the microperimetry results.
o. The intermittent sound will start and using this plus your guidance will help the participant align the selected retinal area with the fixation target
p. Encourage the participant to maintain fixation in the position that triggers the continuous tone for periods of 15 seconds
q. Remind the participant to be aware/remember the position of their eye during the training.

r. Give the participant brief rest periods every 10 minutes

8.5 After biofeedback training

a. Record distance visual acuity using the same eye trained above i.e. measuring acuity using the TRL and keeping the other patched (see Visual Acuity SOP)
b. Hold a pen up and ask the participant to look at it until it goes misty
c. With the participant still looking at the pen, ask them to look at the Bailey Lovie near chart and see if the text is clear (this should be in the lower field and correspond with the TRL)
d. Explain to the participant why this works
e. Repeat step 8.6b-8.6c, but this time use your hand instead of the pen and your face instead of the chart.
f. Ask the participant to look away and repeat 8.6e several times until automatic
g. Record near VA with the Bailey Lovie chart on a clip board with the participant sitting in a comfortable position. Start with the largest size print and work down the card until the participant struggles to read. They can use appropriate near vision spectacle correction and advise them to move the card rather than their eyes. If the participant is struggling to use their TRL, hold a pen up and get them to look at it until it goes misty and simultaneously observe the text, which should then be where the TRL is.
h. Using the Functional Field Test Chart, ask the participant to read the sentences keeping their eyes in a position using the TRL. Observe the participant and note which sentence they make their first eye movement including the number of words.
i. Using Chart 4, choose a size that's similar to near acuity
j. Ask the participant to look at number 5 using the TRL & simultaneously see if they can see numbers 4 & 6, then 3 & 7, then 2 & 8.
k. Once participant understands the TRL concept, repeat 8.6g-8.6j with an appropriate magnifier/hyperoculars. Keep reminding the participant not to move their eye/head/magnifier only slide the book
l. Advise participant to practice little and often – 4x-5x a day looking at bills/letters for about 10mins, as well as completing the homework give from the training
m. Arrange a follow-up appointment for one weeks' time to a total of three visits

8.3 Close down

a. Store all of the equipment.
